# Low risk of SARS-CoV-2 in blood transfusion

**DOI:** 10.1371/journal.pone.0249069

**Published:** 2021-04-13

**Authors:** Michael Owusu, Augustina Angelina Sylverken, Philip El-Duah, Nana Kwame Ayisi-Boateng, Richmond Yeboah, Eric Adu, Jesse Asamoah, Michael Frimpong, Japhet Senyo, Godfred Acheampong, Mohamed Mutocheluh, John Amuasi, Ellis Owusu-Dabo, Yaw Adu-Sarkodie, Richard Odame Phillips

**Affiliations:** 1 Department of Medical Diagnostics, Kwame Nkrumah University of Science and Technology, Kumasi, Ghana; 2 Kumasi Centre for Collaborative Research in Tropical Medicine, Kwame Nkrumah University of Science and Technology, Kumasi, Ghana; 3 Centre for Health Systems Strengthening, Kumasi, Ghana; 4 Department of Theoretical and Applied Biology, Kwame Nkrumah University of Science and Technology, Kumasi, Ghana; 5 Institute of Virology, Charite, Universitätsmedizin Berlin, Berlin, Germany; 6 Department of Medicine, Kwame Nkrumah University of Science and Technology, Kumasi, Ghana; 7 Department of Molecular Medicine, Kwame Nkrumah University of Science and Technology, Kumasi, Ghana; 8 Department of Clinical Microbiology, Kwame Nkrumah University of Science and Technology, Kumasi, Ghana; 9 Department of Global and International Health, Kwame Nkrumah University of Science and Technology, Kumasi, Ghana; University of Cincinnati College of Medicine, UNITED STATES

## Abstract

**Background:**

The novel coronavirus disease (COVID-19) pandemic, caused by severe acute respiratory syndrome coronavirus-2 (SARS-CoV-2), continues to remain a global challenge. There is emerging evidence of SARS-CoV-2 virus found in the blood of patients from China and some developed countries. However, there is inadequate data reported in Ghana and other parts of Africa, where blood transfusion service heavily relies on voluntary and replacement blood donors. This study aimed to investigate whether plasma of infected individuals could pose significant transfusion transmitted risk of COVID-19 in Ghanaian populations.

**Methods:**

This cross-sectional retrospective study was conducted at the Kumasi Centre for Collaborative Research into Tropical Medicine (KCCR), KNUST, Ghana. Study subjects comprised contacts of COVID-19 individuals, those with classical symptoms of COVID-19 and individuals who had recovered based on the new Ghana discharge criteria. Whole blood, sputum or deep coughed saliva samples were collected and transported to KCCR for SARS-CoV-2 testing. Viral nucleic acid was extracted from sputum/nasopharyngeal samples using Da An Gene column based kit and from plasma using LBP nucleic acid extraction kit. Real-Time PCR was performed specifically targeting the ORF1ab and Nucleocapsid (N) genomic regions of the virus.

**Results:**

A total of 97 individuals were recruited into the study, with more than half being males (58; 59.7%). The mean age of all subjects was 33 years (SD = 7.7) with minimum being 22 years and maximum 56 years. Majority (76; 78.4%) of all the subjects were asymptomatic, and among the few symptomatic subjects, cough (10; 10.3%) was the most predominant symptom. Of the 97 sputum samples tested, 79 (81.4%) were positive for SARS-CoV-2. We identified SARS-CoV-2 viral RNA in the plasma of 1 (1.03%) subject who had clinically recovered.

**Conclusion:**

This study reports the identification of SARS-CoV-2 viral RNA in a convalescent individual in Ghana. Due to the low prevalence observed and the marginal cycling thresholds associated, the risk of transfusion transmission of SARS-CoV-2 is negligible. Well-powered studies and advanced diagnostics to determine infectious viremia is recommended to further evaluate the potential risk of hematogenous transmission among recovered patients.

## Introduction

Severe acute respiratory syndrome coronavirus-2 (SARS-CoV-2), is the aetiological agent for COVID-19. The virus continues to spread with more than 66 million infections and over 1.5 million deaths as of 5^th^ December, 2020 [1]. Ghana confirmed her first two cases of COVID-19 on the 12^th^ of March, 2020 and the case counts have risen to 76,573 with 594 deaths as of 27^th^ December, 2020 [2].

Given the rapid spread of the virus and the potential for it to run through the population, it is important to assess how this epidemic could impact on blood transfusion within the African context. A number of studies have reported the presence of SARS-CoV-2 ribonucleic acid [3] (RNA) in plasma, blood and serum of infected people who have been hospitalized [3, 4]. These findings vary depending on the clinical status of the patient. Zhang *et al*., reported the identification of 6 out of 15 hospitalized patients (40%) who were RNA-positive for SARS-CoV-2 in their blood in Wuhan [3]. A recent study in Germany however did not identify the presence of SARS-CoV-2 in blood samples from symptomatic patients with mild symptoms [5]. The only patient identified to have SARS-COV-2 had acute respiratory distress syndrome and was artificially ventilated. A more recent study also identified SARS-CoV-2 RNA in plasma from an eligible donor after 40 days after resolution of symptoms of COVID-19 disease [6].

The blood transfusion service in Ghana mainly relies on voluntary and replacement blood donors. Replacement donors are individuals donating blood to replace what has been transfused to a relative or a friend. All potential blood donors should be between 18 and 65 years. All potential blood donors are screened for HIV, Syphilis and Hepatitis B and also examined to be medically fit. However, with the COVID-19 pandemic, questions arise as to whether SARS-CoV-2 can be transmitted through blood and whether an infected asymptomatic individual or those who have clinically recovered from COVID-19 should be excluded from blood donation. With Ghana’s determination to prepare plasma from recovered COVID-19 patients for transfusion, there is the need to investigate whether blood or plasma donated could pose significant risk of transmission in Ghanaian populations.

## Materials and methods

### Study area

This study was conducted at the Kumasi Centre for Collaborative Research into Tropical Medicine (KCCR). KCCR was established as a joint venture between the stakeholders of Ministry of Health of the Republic of Ghana, Kwame Nkrumah University of Science and Technology, and the Bernhard Nocht Institute of Tropical Medicine (BNITM). KCCR is currently the second largest COVID-19 testing site in the country serving the northern part of Ghana. At the early phase of the pandemic, KCCR received samples from 12 out of 16 regions in Ghana and tested a daily average of 1,200 samples.

### Study design

This was a cross-sectional retrospective study involving contacts of individuals with confirmed COVID-19 and those presenting with signs and symptoms of COVID-19 disease or deemed to have clinically recovered as per the new Ghana Health Service discharge criteria [7]. Recruitment of cases at the facilities was done according to Ghana National Surveillance Strategy protocol [8]. Suspected COVID-19 cases were defined as individuals presenting with fever (>38°C) and symptoms of respiratory tract illness (e.g., cough, shortness of breath) or in close contact with a person who is under investigation or confirmed for COVID-19.

Whole blood, sputum or deep-coughed saliva samples from these individuals were submitted to KCCR for SARS-CoV-2 testing. Plasma was separated from cells by centrifugation and aliquoted into 1.5ml cryotubes for storage at -80°C. Plasma and sputum from deep cough were tested using Real time polymerase chain reaction (RT-PCR) technology. Viral nucleic acid was extracted from the sputum using Da An Gene column based kit (Guangdong, China) following the manufacturer’s protocol. For plasma samples, viral nucleic acid was extracted using LBP nucleic acid extraction kits (Guangzhou LBO Medical Science and Technology Co., Ltd). Briefly, 200 μl of sample was added to 20 μl of proteinase K and 400 μl of Lysate solution. The mixture was transferred into a spin column after incubating for 72°C for 10 mins. The mixture was centrifuged and taken through two steps of washing after discarding the filtrate. The final solution was eluted in 50 μl of elution buffer.

Real-time polymerase chain reaction (RT-PCR) test was performed on samples using Da An Gene PCR kits (Guangdong, China). The PCR kit is designed to target the open-reading frame 1ab (ORF1ab) and Nucleocapsid (N) regions of the virus. A 25 μl reaction volume was used for each PCR reaction and this consisted of 17 μl of solution A (ORF1ab/N), 3μl of solution B (ORF1ab/N) and 5μl of RNA template. The component mixture for solution A and B were thoroughly mixed and centrifuged for a short time before addition of the RNA template Prior to using the kits, standardization and validation for detection of SARS-CoV-2 were performed using positive and negative controls. All extracted RNA samples were tested in duplicate on freshly extracted RNA but this was done different occasions. Positive samples were also re-extracted and re-tested. All tests were run using the CFX 96 Real time PCR machine. The PCR was set up using cycling conditions of 50°C for 15 minutes for Reverse Transcription. Prior to running the samples, validation experiments was performed on the CFX 96 Realtime PCR machines by testing serial dilutions of positive control. The reaction set up was 95°C for 15 minute for denaturation and 45 cycles of 94°C for 15 seconds and 55°C for 45 seconds. The analytical sensitivity of the kits was 500 copies/ml. All samples with cycling threshold of 40 and above were considered as negative. Internal controls were also analyzed for every sample according the manufacturer’s protocol.

### Ethical process

We obtained ethical approval from the Committee on Human Research, Publication and Ethics of the School of Medicine and Dentistry, Kwame Nkrumah University of Science and Technology (KNUST) (CHPRE/AP/462/19) and the Ethics Review Committee of the Ghana Health Service (GHS-ERC087/03/20). Due to the retrospective nature of the study, informed consent was not obtained, and data were analyzed anonymously.

## Results

### Patient characteristics

A total of 97 individuals made of 39 females (40.2%) and 58 males (59.8%) were enrolled into this study. Of the 97 individuals, 79 (81%) were cases and 18 were subjects deemed to have clinically recovered. Of the 79 cases, 58 (73.4%) were asymptomatic and 21 (26.6%) were symptomatic. The clinical presentations recorded among the 21 symptomatic individuals were anosmia (3; 14.3%), cough (10; 47.6%), fever (5; 23.8%) and nausea (3; 14.3%). All samples were collected from patients on the day they presented with COVID-19 like symptom at the hospital. The mean age of all subjects was 33 years (SD = 7.7) with minimum being 22 years and maximum 56 years. Approximately 50.5% (49) of patients were aged 22–32 years and 49.5% (48) were aged 33–56 years. The minimum age of asymptomatic subjects was 23 years and maximum age was 56 years.

### Virus characteristics

#### Sputum/Saliva samples

All sputum or deep coughed saliva samples were processed for SARS-CoV-2. Seventy-eight (78; 80.4%) samples were positive for SARS-CoV-2. Of the 78 positive samples, 57 (73.1%) were asymptomatic and the rest were symptomatic. None of the sputum or saliva samples collected from clinically recovered patients were positive for SARS-CoV-2.

#### Plasma samples

SARS-CoV-2 genomes was detected in only 1 (1.03%) of the 97 blood samples examined. This detection was from a subject who had clinically recovered as per the Ghana protocol. The positive signal showed a cycling threshold of 35.5 for the ORF1ab and 36.14 for the N gene. The time period between onset of symptoms and collection of the blood sample was 23 days. A second sample also showed cycling threshold of 39 for only ORF1ab but negative for the N gene. Repeated testing of fresh samples on different occasions however showed negative probably due to freeze-thawing of samples. This samples was therefore considered as indeterminate. Table 1 gives a brief description of the viruses found in all samples including those from plasma.

**Table 1:** Virus characteristics of participants with suspected COVID-19 providing either blood or sputum samples.

Total	Sputum/saliva samples		Plasma samples	
	Negative	Positive	Negative	Positive
	19	78	95	1
Symptoms of covid-19 n (%)				
Anosmia	0 (0)	3 (3.8)	3 (3.2)	0 (0)
Asymptomatic	19 (100)	57 (73.1)	74 (77.9)	1 (100)
Cough with flu-like symptoms	0 (0)	10 (12.8)	10 (10.5)	0 (0)
Fever with flu-like symptoms	0 (0)	5 (6.4)	5 (5.3)	0 (0)
Nausea	0 (0)	3 (3.8)	3 (3.2)	0 (0)
Gender of subjects n (%)				
Females	6 (31.6)	33 (42.3)	39 (41.1)	0 (0)
Males	13 (68.4)	45 (57.7)	56 (58.9)	1 (100)
Age groups n (%)				
22–32	7 (36.8)	42 (53.8)	48 (50.5)	1 (50)
33–56	12 (63.2)	36 (46.2)	47 (49.5)	0 (0)

## Discussion

The life-saving role of blood and blood products cannot be overemphasized and is highlighted by the listing of whole blood, red blood cells, platelets and frozen plasma on the World Health Organization (WHO) Model List of Essential Medicines (EML) and the WHO ‘Guidelines on management of blood and blood components as essential medicines [6]. Notwithstanding, in the urgent requirement of blood, it is critical to know whether SARS-CoV-2 could pose significant transfusion transmission risk to African populations. Our study presented data on 97 symptomatic and asymptomatic individuals, 18 of whom had been declared as having recovered clinically according to the Ghana Health Service protocol. SARS-CoV-2 genomes were detected in only 1 (1.03%) of the 97 blood samples examined. The positive sample was detected in a patient who had been declared as having clinically recovered from the virus after 23 days of showing symptoms. Our findings are in line with other published articles which have equally identified SARS-CoV-2 in blood [3, 4, 9]. The persistence of RNAemia after clinical recovery is quite remarkable and of much concern in the light of new guidelines being developed for blood donation. Although the prevalence is low, it’s important for stakeholders involved in crafting donation policies to pay particular attention to possible risk associated with blood donation exercises.

It is however important to state that RNA detection in blood is not equivalent to infectiousness. Hence, we cannot confirm whether the SARS-CoV-2 identified in the sample is able to infect blood tissues when transfused. This is because of our inability to culture or grow the virus due to the lack of logistics at our Centre. RNA from related coronaviruses that cause SARS and Middle East respiratory syndrome (MERS) have also been found in blood and outside the respiratory tract but infectious virus is not described [10–12]. For SARS-CoV-2 to be classified as transfusion-transmitted disease, it must cause illness in the transfused infected recipient. This will require follow up studies on transfused subjects and demonstration of disease causation.

One limitation of this study was our inability to demonstrate presence of the virus in samples upon repeat testing due to freeze-thawing nature of the samples. We did not also perform sequencing to further confirm the presence of this virus in the clinical samples. The findings of this study therefore needs to be interpreted with care because of the inconsistency of RNA detection after follow up testing. RNA are generally unstable hence when subjected to freeze and thawing, they tend to loose their sensitivity. Nevertheless the identification of the virus on first time testing could suggest that the virus might be present in the samples.

## Conclusion

Our study has identified RNAemia in a clinically recovered subject in Ghana. We are unable to tell whether this could pose a transfusion-related risk of transmission since we did not test for infectious viremia. Due to the low prevalence observed and the marginal cycling thresholds associated, the risk of transfusion transmission of SARS-CoV-2 is negligible. Well-powered studies and advanced diagnostics to determine infectious viremia is recommended to further evaluate the potential risk of hematogenous transmission among recovered patients.

## Supporting information

S1 Dataset (XLSX)
